# A Novel Method of Frontal Bone Reconstruction Using Patient-Specific Implants and Costochondral Grafts: A Case Report

**DOI:** 10.7759/cureus.57767

**Published:** 2024-04-07

**Authors:** Shrishty Bhardwaj, Sneha Pendem, Kalyani P, Kathiravan Selvarasu, Murugesan Krishnan

**Affiliations:** 1 Oral and Maxillofacial Surgery, Saveetha Dental College and Hospitals, Saveetha Institue of Medical and Technical Sciences, Saveetha University, Chennai, IND

**Keywords:** post-traumatic residual deformity, patient-specific implant (psi), innovative technique, novel therapies, frontal bone, costochondral graft, cranial reconstruction

## Abstract

The frontal bone is the vital component of the human skull and forms a part of the anterior skull vault, base, and roof of the orbits. Frontal bone defects may arise secondary to various causes like trauma, congenital defects including craniofacial clefts, tumors in the frontal bone requiring surgical intervention, and infections, like osteomyelitis, that cause osteonecrosis of the frontal bone. Reconstruction of frontal bone has been explored in the literature, and various materials are available for rehabilitation, like auto/allografts, and alloplastic materials, including bone cement, titanium meshes, and patient-specific implant (PSI). All the available materials have their own advantages and disadvantages; hence, depending on the anatomy and physiology of the frontal bone and the involvement of the naso-orbito-ethmoidal (NOE) complex, patient selection and treatment plan become very crucial. This report presents a case of the frontal bone with a NOE defect, secondary to trauma, reconstructed using a PSI and costochondral graft.

## Introduction

The frontal bone is the vital component of the human skull and forms a part of the anterior skull vault, base, and roof of the orbits. Frontal bone defects may arise secondary to various causes that may range from trauma, congenital defects including craniofacial clefts, tumors in the frontal bone requiring surgical intervention, and infections, like osteomyelitis, that can cause osteonecrosis of the frontal bone. Iatrogenic defects may present secondary to craniectomy or cranioplasty performed for elevated intracranial pressure, mainly in patients with head injuries [1].

Trauma to the frontal bone is common with faciomaxillary injuries and has a detrimental effect on facial esthetics and function. Considering its role in the protection of the brain, injuries to the frontal bone can have poor neurologic outcomes, including death due to injuries involving the superior sagittal sinus, leading to life-threatening hemorrhage and air embolism. The aesthetics of the face also become another important concern that warrants precise management of the injuries to the frontal bone [2].

Injuries of the frontal bone constitute about five to 15% of injuries to the facial skeleton. The rarity of these injuries is attributed to the position in the facial skeleton and the thicker cortex with minimal cancellous bone. Often, these lead factors lead to the greater quantum of force required to fracture the same. Hence, high-velocity injuries often lead to the involvement of the frontal bone in trauma and often require complex planning for management [3].

The presence of air sinuses further increases the complexity of management, with a higher propensity for infection. Thus, often these injuries are addressed at a later date as secondary procedures unless functional demands warrant primary management [4]. Secondary deformity management of frontal bone injuries needs the use of auto/ allografts to correct the esthetic deformity. Alloplastic materials are the choice for management for most surgeons considering the large volume available, ease of working, and lack of donor site morbidity. Various allografts are commonly used, including bone cement, titanium meshes [5], or patient-specific implants (PSI) [6,7]. Each of these materials has its own set of merits and demerits. The current paper aims to describe a post-traumatic secondary deformity of the frontal bone and the naso-orbito-ethmoidal (NOE) complex reconstructed with a PSI and rib graft.

## Case presentation

**Figure 1:**
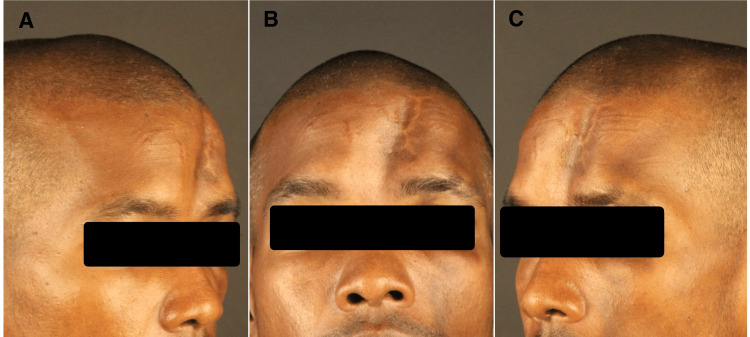
Preoperative extra-oral photos A: left oblique, B: frontal, C: right oblique

**Figure 2:**
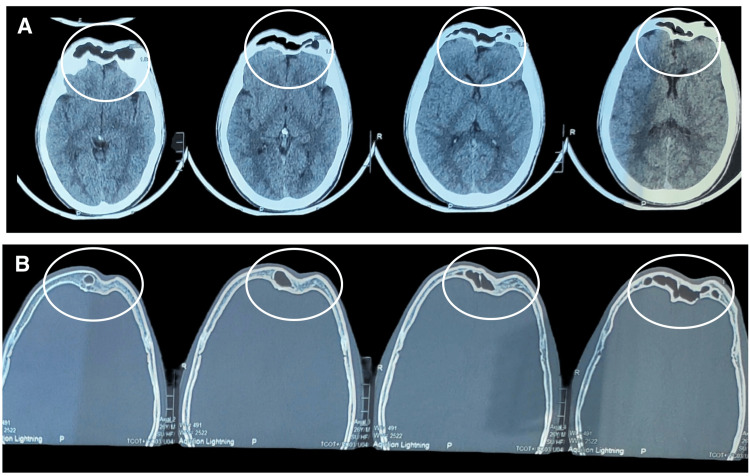
CT films depicting a fracture of the frontal bone A: soft tissue section, B: hard tissue section CT: computed tomography

A 24-year-old male patient reported to our department with complaints of esthetic concern secondary to depression in his forehead region (Figure 1). History revealed a road traffic accident three years ago with a frontal impact leading to a head injury. Medical records revealed the patient had displaced comminuted frontal bone (anterior comminuted and posterior minimally displaced) with NOE complex fracture (Figure 2) and cerebrospinal fluid rhinorrhea. The patient was under conservative management for the same. Reconstruction of the frontal bone and NOE complex fracture for correction of esthetic deformity were deferred to a later date.

After three years, the patient presented to us with a frontal bone defect measuring 33 mm in width and 27 mm in length, with traumatic telecanthus (intercanthal distance of 38 mm), a depressed nasal bridge, and paresthesia of the forehead with saddle nose deformity. There were no neurological deficits, and the ophthalmic exam was normal. CT evaluation revealed malunited frontal (both the anterior and posterior frontal bone tables) and NOE complex fractures. The intercanthal distance was 38 mm (minimally increased); however, due to the fracture of the NOE region, an exaggerated intercanthal distance was perceived, suggesting a pseudo-telecanthus. This was addressed by augmentation of the nasal dorsum, a camouflage procedure to correct the pseudo-telecanthus.

Considering there were no functional deficits and only esthetic concerns were present, the patient was planned for the reconstruction of the frontal bone deformity with a titanium PSI and costochondral graft for the correction of the NOE complex.

Designing of the PSI

**Figure 3:**
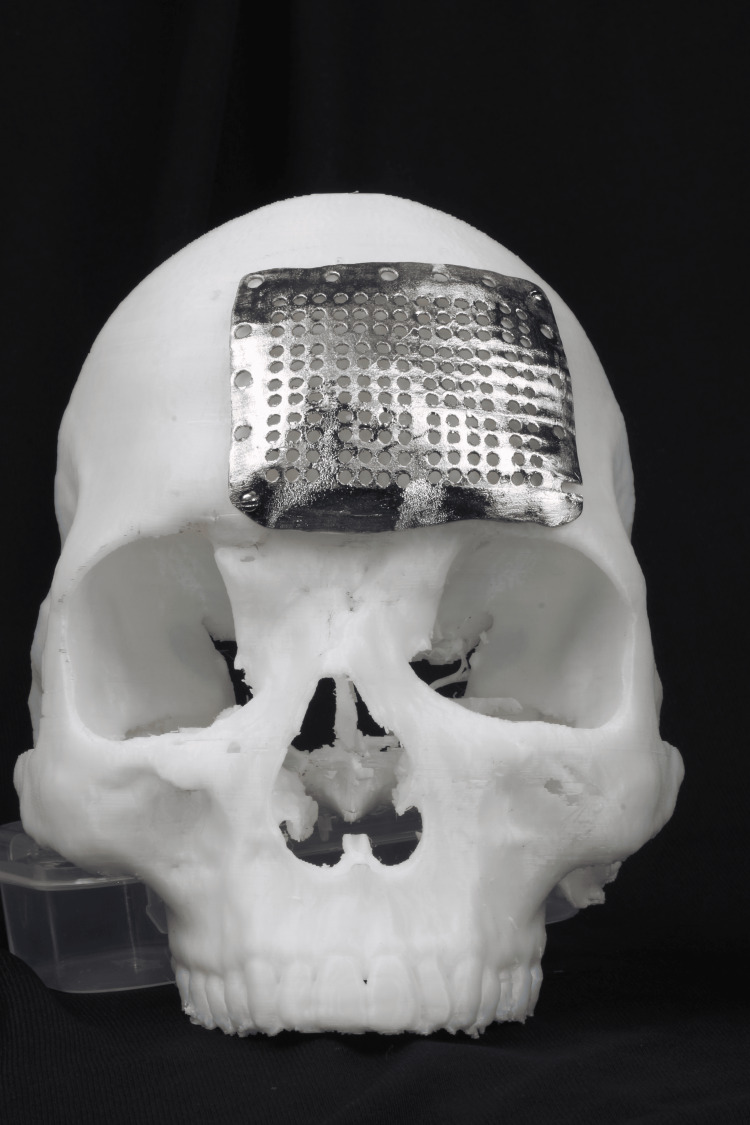
PSI with skull model PSI: patient-specific implant

After all necessary investigations, PSI planning was done. Digital Imaging and Communications in Medicine files derived from the CT scan were converted to stereolithography (STL) format and printed for a three-dimensional view of the defect. A titanium patient-specific frontal mesh was designed using Freeform and Geomagic software (3D Systems, Rock Hill, South Carolina, USA). The mesh, which was 0.8 mm thick, was designed with 0.5 mm holes throughout, and 1.2 mm holes were made around the periphery for the fixation of screws. In the present case scenario, PSI was designed to extend onto the radix of the nose to facilitate stabilization of the costochondral graft; hence, PSI was our choice. Apart from this, good contour to the forehead was possible, which was facilitated by the rigidity of the PSI. This cannot be effectively achieved by stock titanium meshes (Figure 3).

Surgical procedure

Incision and Dissection

**Figure 4:**
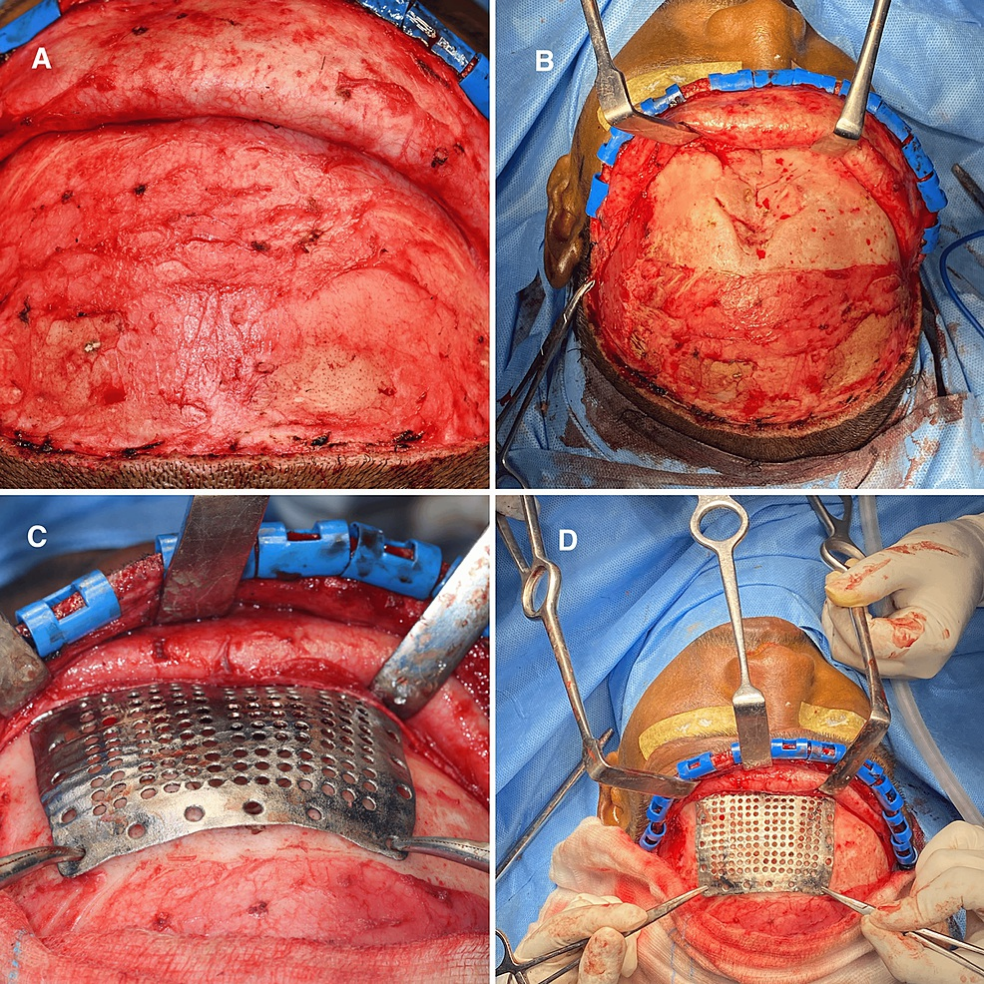
Exposure of the defect and placement of PSI A: subgaleal plane, B: exposure of the defect, C and D: placement of PSI PSI: patient-specific implant

The defect was approached through the standard coronal approach, where the incision was placed in the hairline. The flap was elevated in the subgaleal plane, and the periosteum was elevated from the region of the hairline to expose the frontal defect and the root of the nose. Additionally, a hemi transfusion incision was placed in the membranous septum, and dissection was done until the nasal dorsum to create a tunnel and harbor the cartilaginous dorsal strut (Figure 4).

Placement of PSI

**Figure 5:**
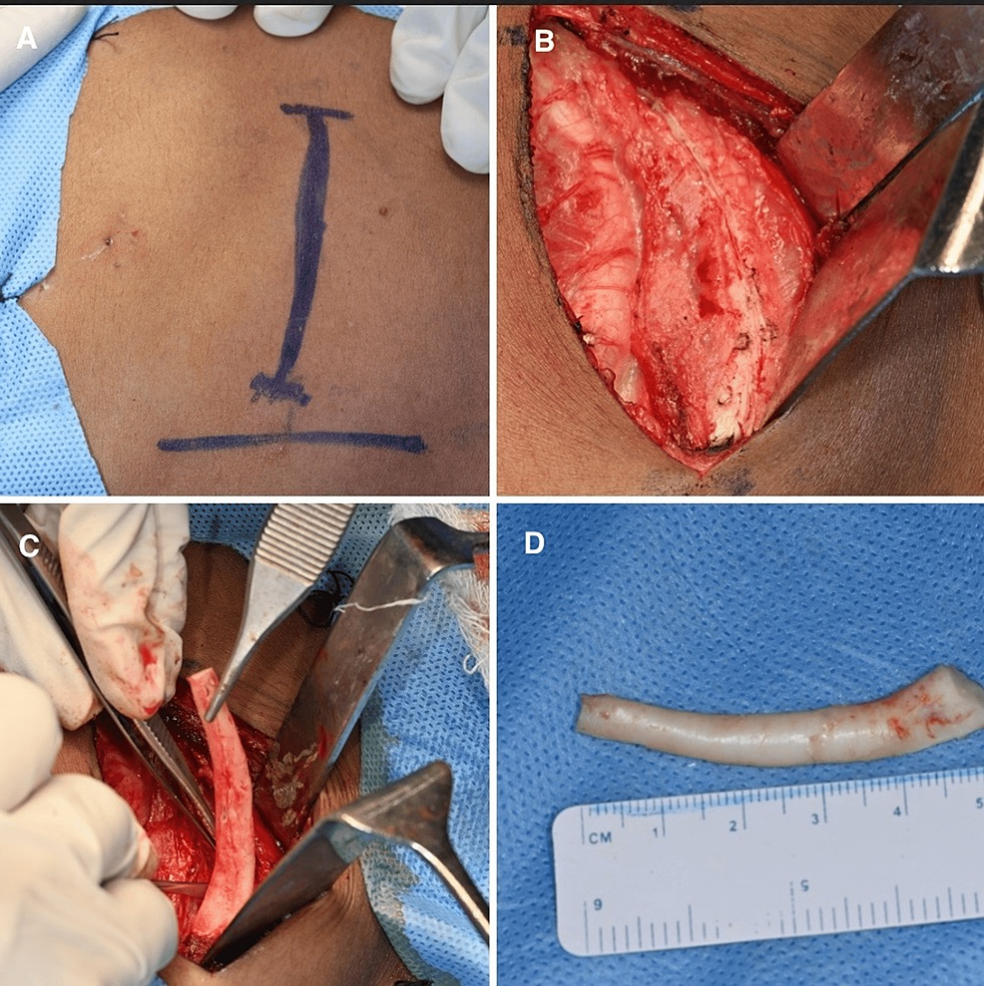
Harvest of costochondral graft from the 10th rib A: incision marking, B: flap elevation and exposure, C: 10th rib, D: harvest of costochondral graft

**Figure 6:**
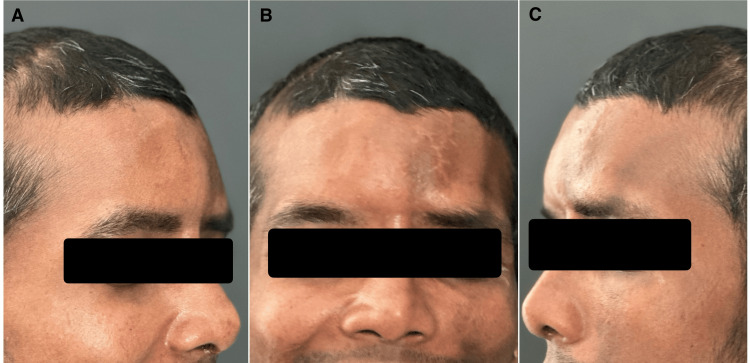
Post-operative extra-oral photo A: left oblique, B: frontal, C: right oblique

The PSI was placed, and position and fit were checked and confirmed. Cartilaginous rib graft that was harvested from the 10th rib (6 cm x 1 cm) and was carved after scoring the periosteum (Figure 5). The graft was placed in the nasal dorsal tunnel to reconstruct the nasal dorsum. The PSI was stabilized using titanium 1.5 mm self-drilling screws of 5mm length (Figure 6).

Incisions were closed in layers to achieve water-tight closure with active drains in place. Extubation was uneventful with a stable post-operative course, and a two-month follow-up of the patient revealed no untoward sequelae (Figure 7).

## Discussion

Reconstruction of facial skeleton needs is often challenging considering the esthetic and functional demands of the craniofacial skeleton. The frontal bone is a vital component of the cranial vault that protects the brain and forms the roof of the orbit. It encloses the frontal sinus, which drains into the middle meatus of the nasal cavity. These injuries to the frontal bone can have detrimental effects on the esthetics and functioning of the vital organs, including the brain and the globe of the eye [8].

Reconstruction of the frontal and the NOE complex can be challenging and will often require precise planning to satisfy the structural and functional demands. Primary reconstruction, though appealing, is often deferred considering the involvement of head injuries and global injuries in trauma. Apart from this, the comminution of the anterior table and the NOE complex may demand the use of autogenous or alloplastic grafts that carry the risk of infection, which can progress to meningeal infections that can have dire complications of meningitis. To overcome these issues, secondary reconstruction is often opted for by surgeons unless functional demands warrant primary reconstruction [9].

The primary objective of secondary reconstruction includes the restoration of facial esthetics without disrupting the functioning of the craniofacial skeleton. Re-osteotomies and only grafting are the popular modes for secondary reconstruction. Re-osteotomies of the NOE complex and frontal bone are often not opted for, as the bones of the medial orbital wall that constitute the NOE complex are extremely thin and have a greater propensity for avascular necrosis during re-osteotomies. A similar situation may be seen with anterior table fractures, and re-osteotomies of the frontal bone may also be associated with displacement of the posterior table, which can lead to the recurrence of cerebrospinal fluid leakage and hemorrhage from the superior sagittal sinus. Hence, only grafting to restore contour deformities is the choice of surgical procedure in patients undergoing reconstruction of the frontal and NOE complex fractures [10].

Various autogenous and alloplastic materials have been recommended for the reconstruction of the facial skeleton. However, the choice of reconstructive material to be used depends on the ease of manipulation, cost, biocompatibility, and rejection rates. Various autogenous and alloplastic materials have been used for the reconstruction of frontal bone defects. Calvarial bone from the parietal bone is the choice for smaller defects and is often considered the first option considering the proximity of the donor site to the recipient site, and a single incision is often sufficient for harvest and reconstruction. Rib graft and iliac graft are other choices of donor sites for bone harvest; however, the need for additional donor sites, functional compromise, and higher complication rates are the reasons for surgeons to opt for alloplastic replacements for frontal bone reconstruction [11].

Various alloplastic biomaterials are being used to accomplish frontal bone reconstruction. Self-cure/heat-cure polymethylmethacrylate (PMMA) bone cement was the common choice for frontal bone reconstruction. Later on, polyethylene mesh came into use. The issues with the use of these materials include leaching of material (self-cure PMMA), lack of adequate strength, and warranted impregnation of titanium mesh in the implants. Various alloplastic materials that have been applied for the reconstruction of the facial skeleton are tabulated in Table 1 with their merits and demerits [12].

**Table 1:** Alloplastic materials used for reconstruction PMMA: polymethylmethacrylate; 3D: three-dimensional

Material	Advantages	Disadvantages
Polymethylmethacrylate (PMMA)	Biocompatible acrylic material, strength, radiolucency, and low-cost prefabricated PMMA are porous and permit fibrovascular ingrowth.	Brittleness, inlay implant, requires additional mini plates and screws for fixation and increases the risk of hardware loosening and implant failure.
Polyether ether ketone (PEEK)	Radiolucent and hypodense can be trimmed and re-contoured as required. Lower post-operative infection rate. There is no need for additional hardware.	High cost, presurgical planning, inlay implant, and does not accommodate post-cranioplasty cerebral edema.
Preformed titanium mesh	Biocompatible, various contours and sizes are available, and they are secured directly to the bone. It accommodates dural tacking sutures when indicated to reduce extradural dead space and robust vascularity from soft tissue ingrowth.	Extrusions can occur in patients with composite defects. Hyperdense on the radiograph causes significant artifacts.
3D-printed custom titanium implants	Predictable fit, ideal for large defects due to their rigidity and stability, reduces the risk of dehiscence.	High cost, not suitable for immediate or early reconstruction.
Porous polyethylene (MEDPOR)	Malleable and moldable while maintaining structural rigidity.	Increased chances of infection.

Considering the aforementioned, titanium implants are seen as the best choice of reconstructive material for defects of the frontal bone. Stock titanium implants may need more precision in the design required for the specified defect of the craniofacial skeleton. To overcome the same, designing a PSI that is customized to precisely the defect is the current choice of treatment. Computer-aided design facilitates variability in the thickness and design as per the patient's needs, which are adapted to the STL model preoperatively to reduce the intra-operative time. Considering the PSI to be made from titanium alloy, no adverse reactions have been noticed so far in our case.

## Conclusions

PSI represents a valuable tool in frontal bone reconstruction, offering personalized solutions with enhanced precision and outcomes. While considerations such as cost and lead time exist, the benefits of improved cosmesis, stability, and reduced surgical complexity make PSIs a promising option for patients requiring frontal bone reconstruction.

## References

[1] Marinheiro BH, de Medeiros EH, Sverzut CE, Trivellato AE (2014). Frontal bone fractures. J Craniofac Surg.

[2] Dumitru M, Vrinceanu D, Banica B, Cergan R, Taciuc IA, Manole F, Popa-Cherecheanu M (2022). Management of aesthetic and functional deficits in frontal bone trauma. Medicina (Kaunas).

[3] Barca I, Stroscio C, Cordaro R, Boschetti CE, Della Torre A, Cristofaro MG (2021). Reconstruction of comminuted frontal bone fracture with titanium plates and acrylic resin: report of two cases. Interdiscip Neurosurg.

[4] Gerbino G, Roccia F, Benech A, Caldarelli C (2000). Analysis of 158 frontal sinus fractures: current surgical management and complications. J Craniomaxillofac Surg.

[5] Chattopadhyay C (2019). Reconstruction of acquired frontal bone defects using titanium mesh implants: a retrospective study. J Maxillofac Oral Surg.

[6] Manrique OJ, Lalezarzadeh F, Dayan E, Shin J, Buchbinder D, Smith M (2015). Craniofacial reconstruction using patient-specific implants polyether ether ketone with computer-assisted planning. J Craniofac Surg.

[7] Camarini ET, Tomeh JK, Dias RR, da Silva EJ (2011). Reconstruction of frontal bone using specific implant polyether-ether-ketone. J Craniofac Surg.

[8] Machtens E, Lausberg G, Stursberg W (1979). Principal considerations in the reconstruction of frontal-bone defects and frontal-bone dislocations following injuries (Article in German). Fortschr Kiefer Gesichtschir.

[9] Singh O, Varacallo M (2024). Anatomy, Head and neck: frontal bone. StatPearls [Internet].

[10] Kim TW, Kim MJ (2022). Application of one piece autologous rib cartilage graft in hollow space of complete Naso-ethmoid orbital fracture. J Craniofac Surg.

[11] Gentile P, Cervelli V (2009). Nasal dorsum reconstruction with 11th rib cartilage and auricular cartilage grafts. Ann Plast Surg.

[12] Park EK, Lim JY, Yun IS, Kim JS, Woo SH, Kim DS, Shim KW (2016). Cranioplasty enhanced by three-dimensional printing: custom-made three-dimensional-printed titanium implants for skull defects. J Craniofac Surg.

